# Veno-occlusive disease/sinusoidal obstruction syndrome in patients with prior gemtuzumab ozogamicin: literature analysis of survival after defibrotide treatment

**DOI:** 10.1038/s41408-020-0286-5

**Published:** 2020-03-04

**Authors:** Paul G. Richardson, Selim Corbacioglu

**Affiliations:** 1 Jerome Lipper Multiple Myeloma Center, Dana-Farber Cancer Institute, Harvard Medical School, Boston, MA USA; 2 Department of Pediatric Hematology, Oncology and Stem Cell Transplantation, University of Regensburg, Regensburg, Germany

**Keywords:** Haematological cancer, Cancer

Dear Editor,

Hepatic veno-occlusive disease/sinusoidal obstruction syndrome (VOD/SOS) is a potentially life-threatening complication of hematopoietic cell transplantation (HCT) conditioning or non-transplant-associated chemotherapy^1^. The three established symptoms of VOD/SOS are elevated bilirubin (although ~20% of patients have anicteric VOD/SOS), sudden weight gain (ascites), and hepatomegaly/liver tenderness^1^. Severe, untreated VOD/SOS has been reported to have a mortality rate >80%, and can result in multi-organ dysfunction (MOD), typically renal and/or pulmonary dysfunction^1^.

VOD/SOS is associated with endothelial cell (EC) damage from chemotherapy and high-dose HCT-conditioning regimens^2^. During HCT, ECs are activated and damaged by cytokines produced by injured tissues and toxic chemotherapy metabolites. EC dysfunction leads to loss of cytoskeletal structure, inflammatory responses resulting in sinusoidal narrowing, and a shift to a procoagulant and hypofibrinolytic state. These effects reduce hepatic venous outflow and induce post-sinusoidal hypertension, potentially leading to MOD.

The incidence of VOD/SOS in adults ranges from 8% to 14%^2^; it can be influenced by multiple factors, including age, primary disease, diagnostic criteria, conditioning regimen, and type of HCT, which may explain variations in the reported incidence among published studies.

## Risk factors

Multiple factors are known to increase the risk of developing VOD/SOS. Age, leukemia diagnosis, Karnofsky index <90%, *glutathione S-transferase Mu 1* null genotype, platelet refractoriness, sepsis pre-HCT, and pre-existing hepatic or pulmonary dysfunction are all patient-related factors associated with a higher VOD/SOS risk^2^. Prior treatments shown to increase VOD/SOS risk include abdominal radiation, HCT (particularly allogeneic and unrelated/human leukocyte antigen mismatch HCT), high-intensity conditioning regimens, and certain regimens for graft-versus-host disease prophylaxis^2^. Prior treatment with the antibody–drug conjugates gemtuzumab ozogamicin (GO) or inotuzumab ozogamicin (InO) has also been shown to increase the risk of VOD/SOS. The reported odds ratio for developing VOD/SOS following GO exposure is 19.8^2^; based on data from Kantarjian et al.^3^, the odds ratio for VOD/SOS following InO treatment is calculated to be 22.0.

## GO background

GO is a humanized anti-CD33 monoclonal antibody conjugated to calicheamicin, a cytotoxic agent^4^. In 2000, GO was granted accelerated approval by the United States (US) Food and Drug Administration (FDA) for relapsed acute myeloid leukemia (AML) in patients aged >60 years or ineligible for intensive induction chemotherapy. In the first year after approval, a black box warning was added regarding severe or fatal VOD/SOS^5^. In 2010, GO was withdrawn from the US and European markets after a phase 3 study; SWOG S0106 failed to show improved efficacy versus standard of care^5^. Later, the phase 3 ALFA-0701 study demonstrated that a lower, fractionated dose allowed for safer delivery of higher cumulative GO doses (VOD/SOS reported in 6/131 [5%] patients) and led to improved outcomes in patients^6^. Based on these results, GO was reapproved in 2017 by the FDA for the treatment of newly diagnosed and relapsed/refractory CD33-positive AML^4^. In 2018, the European Medicines Agency approved GO combined with daunorubicin/cytarabine for the treatment of patients aged >15 years with de novo CD33-positive AML, except acute promyelocytic leukemia^7^.

The current black box warning for GO lists the risk of hepatotoxicity and VOD/SOS in adult patients who receive higher doses of GO monotherapy, in patients with moderate or severe hepatic impairment prior to receiving GO, and patients treated with GO before or after HCT^4^.

InO also uses calicheamicin as its cytotoxic moiety; it targets CD22 and has been associated with a similar increase in risk of hepatotoxicity and VOD/SOS^3^.

## Defibrotide background

Defibrotide is approved for the treatment of VOD/SOS with renal or pulmonary dysfunction post-HCT in the US and Canada, and severe hepatic VOD/SOS post-HCT in patients aged >1 month in the European Union^8–10^. In vivo evidence suggests defibrotide protects ECs and restores the thrombo-fibrinolytic balance^2^. Data on the response to defibrotide in patients who developed VOD/SOS following treatment with GO are limited. We conducted a literature analysis to evaluate outcomes in patients treated with defibrotide after prior GO exposure.

## Literature analysis

In May 2019, PubMed was searched for studies and case reports to date that included “gemtuzumab ozogamicin” and “defibrotide”. The search included reports on outcomes of defibrotide prophylaxis or treatment for VOD/SOS that developed following GO treatment. Duplicate studies, reviews, or guidelines were excluded.

Overall, 11 publications were identified (Supplementary Fig. 1)^11–21^; 3 were guideline publications or review articles and were excluded from the analysis. The remaining publications were included and comprised four clinical studies, three case reports, and one retrospective study.

The definition of “successful treatment” varied among the identified studies; therefore, the descriptors used for successful treatment (e.g., survival and/or response) were according to each study design.

## Results

### Summary of selected studies

Across the studies, 18 patients received defibrotide prophylaxis following GO exposure (Table 1). One patient who received defibrotide prophylaxis *and* later received defibrotide for treatment of VOD/SOS was also included in the treatment group.

**Table 1:** Summary of examined studies.

Reference	Patient population receiving GO	DF as prophylaxis		Outcome of VOD/SOS with DF treatment
		Number of patients	Incidence of VOD/SOS	
**PubMed search**				
Battipaglia et al. 2017^14^	Retrospective; HCT in 146 adults	4 patients	2 developed VOD/SOS	VOD/SOS treated with DF in 2 patients; both survived (1 received prophylaxis with DF, the other received heparin)
Richardson et al. 2010^15^	20 adult and pediatric patients with VOD/SOS and MOD	—	—	All received DF (phase 2 dose-finding study); Day 100 survival was 50% (*n* = 10/20); trend toward higher CR rate in GO subgroup
Zwaan et al. 2010^16^	30 pediatric patients treated for AML relapse	8 patients	No cases of VOD/SOS	—
Bornhäuser et al. 2008^17^	31 patients with refractory AML	—	—	1 case of VOD/SOS after HCT; treated successfully with DF
Lannoy et al. 2006^18^	1 patient treated for relapsed AML	—	—	DF deemed effective in treating symptoms of VOD/SOS; death attributed to GO failure
Reinhardt et al. 2004^19^	12 pediatric patients treated for relapsed AML	—	—	1 patient with VOD/SOS treated successfully with DF
Versluys et al. 2004^20^	7 patients treated for relapsed AML prior to HCT	6 patients	No cases of VOD/SOS	1 patient without DF prophylaxis had severe VOD/SOS, which was successfully treated with DF
Saviola et al. 2003^21^	1 adult with refractory AML	—	—	VOD/SOS treated successfully with DF
**Congress report identified separately from PubMed search**				
Corbacioglu et al. 2015^22^^a^	16 pediatric patients at high risk of VOD/SOS prior to HCT	11 patients	2 developed VOD/SOS	—

^a^This ASH abstract reporting a study of 356 patients was not included in the analysis, which focused on published manuscripts in PubMed.

*GO* gemtuzumab ozogamicin, *DF* defibrotide, *VOD/SOS* veno-occlusive disease/sinusoidal obstruction syndrome, *HCT* hematopoietic cell transplantation, *MOD* multi-organ dysfunction, *CR* complete response, *AML* acute myeloid leukemia.

A total of 248 patients in the identified studies had been treated with GO, with 36 (15%) patients developing VOD/SOS (Table 1)^14–22^. Of the patients who developed VOD/SOS following GO exposure, 27 were treated with defibrotide.

Additionally, a congress report was analyzed separately from the results of the PubMed search^22^. In that report, a total of 11 patients received defibrotide prophylaxis following GO exposure.

### Defibrotide prophylaxis following GO exposure

**Fig. 1:**
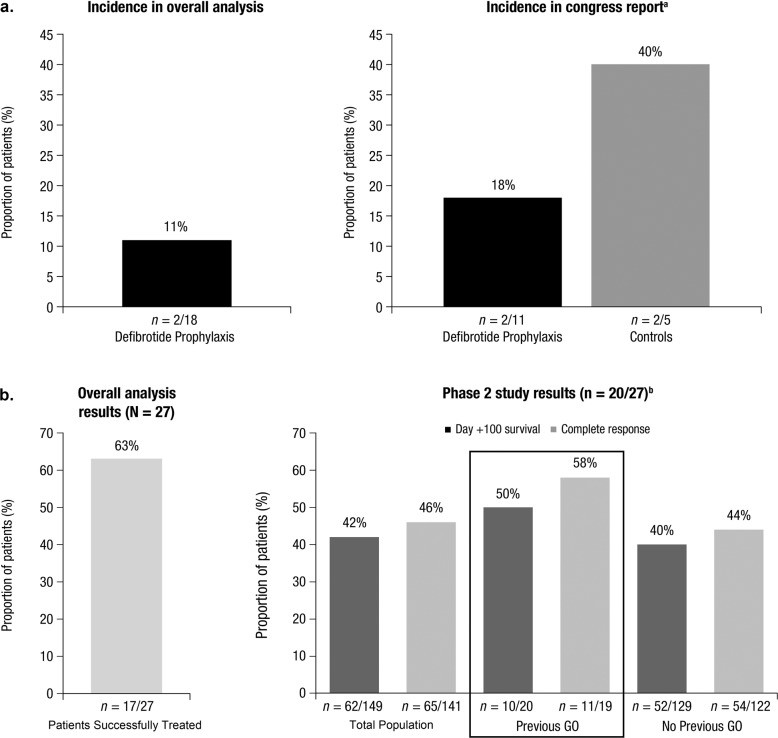
Incidence of VOD/SOS after defibrotide prophylaxis (**a**) and proportion of patients with successful outcomes with defibrotide treatment (**b**) in patients with prior GO exposure. This figure shows the incidence of VOD/SOS in patients receiving defibrotide prophylaxis after GO exposure in the overall analysis and congress report, along with the efficacy of defibrotide in patients with VOD/SOS after GO exposure in the overall analysis and in patients with VOD/SOS receiving defibrotide in the phase 2, dose-finding study with and without GO exposure. VOD/SOS veno-occlusive disease/sinusoidal obstruction syndrome, GO gemtuzumab ozogamicin. ^a^The congress report was analyzed separately from the PubMed search^22^. ^b^A phase 2, dose-finding study investigating defibrotide in VOD/SOS patients post-HCT included 20 (74%) of the 27 patients identified in the overall analysis as receiving defibrotide for VOD/SOS^15^.

Of the 18 patients who received defibrotide for VOD/SOS prophylaxis following GO exposure, 2 (11%) subsequently developed VOD/SOS (Fig. 1a**)**.

In the congress report (analyzed separately), 2 of 11 (18%) patients who received defibrotide prophylaxis for VOD/SOS subsequently developed VOD/SOS (Fig. 1a)^22^. Comparatively, 2 of 5 (40%) control patients who did not receive defibrotide prophylaxis also developed VOD/SOS.

### Defibrotide treatment of VOD/SOS following GO exposure

A total of 27 of 248 (11%) patients across the identified studies developed VOD/SOS following GO exposure and were treated with defibrotide. Treatment was successful (survival and/or response) in 17 of 27 (63%) patients (Fig. 1b). One patient responded to defibrotide but died due to disease progression after failing to respond to GO.

Of the 27 patients in the overall analysis, 20 were from a phase 2, dose-finding study investigating defibrotide in VOD/SOS patients with MOD post-HCT who had prior GO exposure (Fig. 1b)^15^. Ten (50%) of these patients survived to Day 100 post-HCT and 11 of 19 (58%) evaluable patients achieved a complete response (CR). For comparison, patients in the phase 2 study who received defibrotide for VOD/SOS with MOD but had not received previous GO treatment had an overall Day 100 survival rate of 40% (*n* = 52/129) and a CR rate of 44% (*n* = 54/122; Fig. 1b). In the entire study population, the overall Day 100 survival rate was 42% (*n* = 62/149) and the CR rate was 46% (*n* = 65/141; Fig. 1b). As another point of comparison, in a phase 3 study in which only 1 patient in the defibrotide arm had previous exposure to GO, the observed Day 100 survival rate post-HCT in patients treated with defibrotide (*n* = 102) was 38% (95% CI: 29%-48%); in the historical control group (*n* = 32), the observed Day 100 survival rate was 25% (95% CI: 10%–40%)^23^.

Across the studies selected for this analysis, there were no new safety signals identified with defibrotide treatment.

## Discussion

Several studies and analyses have noted the development of VOD/SOS, both post-HCT and without HCT, in patients with prior GO exposure^2,5^. Although the data in the literature are limited, this analysis suggests the efficacy of defibrotide in patients with VOD/SOS post-HCT with prior GO exposure was similar to that observed in VOD/SOS patients without prior GO exposure. Of note, the observed Day 100 post-HCT survival rate of 50% in defibrotide-treated VOD/SOS patients with previous GO exposure compares favorably to the survival rates observed in the overall populations of phase 2 and 3 studies of defibrotide (42% and 38%, respectively).

No new safety signals were identified by this analysis. The safety of defibrotide following GO treatment was comparable to the safety profile reported in previous defibrotide studies.

Similar to GO, patients receiving InO are at a higher risk of developing VOD/SOS^3^. A PubMed search for patients who received defibrotide for the treatment of VOD/SOS following exposure to InO identified three studies^3,24,25^. In these studies, a total of 25 patients who developed VOD/SOS following InO treatment received defibrotide. Resolution of VOD/SOS was not reported in 4 (16%) of these patients as VOD/SOS was ongoing at the time of publication. Among the 21 patients for whom resolution of VOD/SOS was reported, VOD/SOS was resolved in 10 (48%) patients. These observations suggest that, similar to its effect in patients treated with GO, defibrotide may benefit patients with prior InO exposure who develop VOD/SOS post-HCT.

The GO analysis was limited by the small number of studies that reported on outcomes in defibrotide-treated patients with prior GO exposure and the limited number of patients who received defibrotide following GO treatment within those studies. The interpretation of these results is also restricted by a lack of controls, differences in response assessment between studies, the time between GO treatment and transplantation, and the retrospective nature of this analysis.

## Supplementary information

Supplementary Information
