# Supplementary material for: Veno-occlusive disease/sinusoidal obstruction syndrome in patients with prior gemtuzumab ozogamicin: literature analysis of survival after defibrotide treatment
Source: Blood Cancer J. 2020 Mar 4;10(3):29. doi: 10.1038/s41408-020-0286-5 (PMC7055222; doi:10.1038/s41408-020-0286-5)
Supplement: Supplementary file 1 — Supplementary Information [file 41408_2020_286_MOESM1_ESM.docx]

**Supplementary Information**

**Supplementary Figure 1. Study selection**


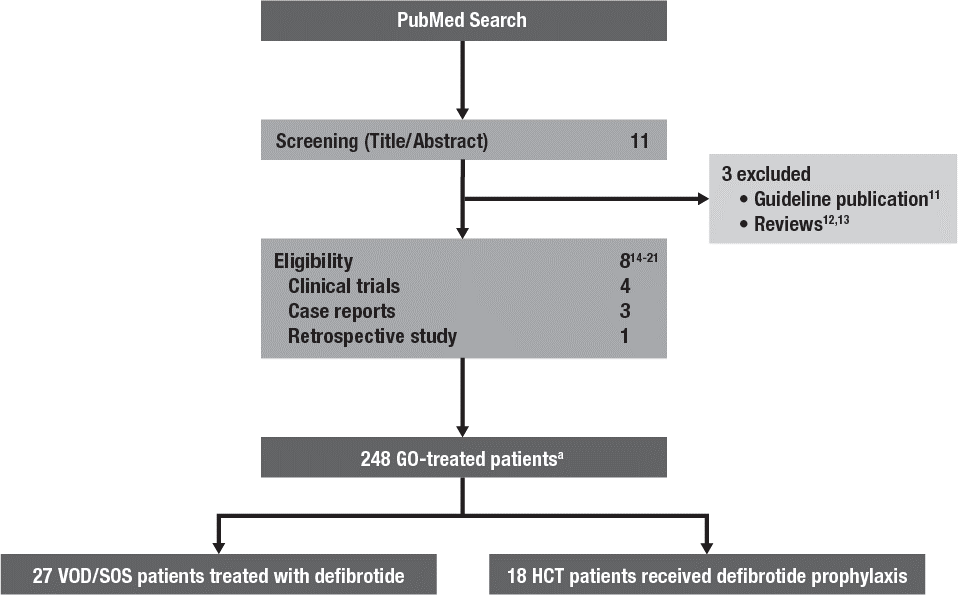


GO, gemtuzumab ozogamicin; HCT, hematopoietic cell transplantation; VOD/SOS, veno-occlusive disease/sinusoidal obstruction syndrome.

^a^15% (36/248) of patients developed VOD/SOS; 1 patient given defibrotide prophylaxis is also included in the treatment group.
